# Cross-talks in colon cancer between RAGE/AGEs axis and inflammation/immunotherapy

**DOI:** 10.18632/oncotarget.27990

**Published:** 2021-06-22

**Authors:** Annachiara Mollace, Maria Laura Coluccio, Giuseppe Donato, Vincenzo Mollace, Natalia Malara

**Affiliations:** ^1^Department of Health Sciences, Research Centre IRC-FSH, University Magna Græcia of Catanzaro, 88100 Catanzaro, Italy; ^2^Department of Experimental and Clinical Medicine, Bionem Laboratory, Magna Græcia University of Catanzaro, 88100 Catanzaro, Italy; ^3^Department of Health Sciences, University Magna Græcia of Catanzaro, Campus S. Venuta, 88100 Catanzaro, Italy; ^*^These authors contributed equally to this work

**Keywords:** RAGE, AGEs, colon cancer, microenvironment, Warburg effect

## Abstract

The tumour microenvironment is the result of the activity of many types of cells in various metabolic states, whose metabolites are shared between cells. This cellular complexity results in an availability profile of nutrients and reactive metabolites such as advanced glycation end products (AGE). The tumour microenvironment is not favourable to immune cells due to hypoxia and for the existence of significant competition between various types of cells for a limited nutrient pool. However, it is now known that cancer cells can influence the host's immune reaction through the expression and secretion of numerous molecules. The microenvironment can therefore present itself in different patterns that contribute to shaping immune surveillance. Colorectal cancer (CRC) is one of the most important causes of death in cancer patients. Recently, immunotherapy has begun to give encouraging results in some groups of patients suffering from this neoplasm. The analysis of literature data shows that the RAGE (Receptor for advanced glycation end products) and its numerous ligands contribute to connect the energy metabolic pathway, which appears prevalently disconnected by mitochondrial running, with the immune reaction, conditioned by local microbiota and influencing tumour growth. Understanding how metabolism in cancer and immune cells shapes response and resistance to therapy, will provide novel potential strategies to increase both the number of tumour types treated by immunotherapy and the rate of immunotherapy response. The analysis of literature data shows that an immunotherapy approach based on the knowledge of RAGE and its ligands is not only possible, but also desirable in the treatment of CRC.

## INTRODUCTION

In recent years, new mechanisms have been discovered and elucidated that link carcinogenesis with cellular metabolism in colon cancer. Despite these progresses, 25% of CRC patients are diagnosed at an advanced stage with a 5-year survival < 20%, and only limited targeted therapies are available [1]. For these reasons, it is necessary to understand comprehensively the underlying mechanisms that promote CRC progression. In particular, the importance of the action of AGEs on their cell receptors here is highlighted to suggest potential new interesting therapeutic targets.

## MOLECULAR AND METABOLIC CHARACTERISTICS OF COLORECTAL CANCER

### 1-MAPK pathway

Most colorectal cancers result from the transformation and progression of precursors such as adenoma. From a molecular point of view, three main pathways of carcinogenesis have been identified: chromosomal instability (CIN), microsatellite instability (MSI) and the CpG island methylator phenotype (CIMP). 85% of CRCs develop from the CIN pathway and have alterations in structure and number of the chromosomes, leading to abnormalities such as aneuploidy, chromosomal rearrangement and loss of heterozygosity at the suppressor gene site. Tumours of the CIN pathway also acquire mutations in oncogenes and tumour suppressor genes including Adenomatous Polyposis Coli (APC), KRAS Proto-Oncogene, BRAF Proto-Oncogene and Tumour suppressor TP53. According to the model of Fearon and Vogelstein.

[2] APC inactivation occurs first in the proliferating epithelium. The most relevant pathways involved in CIN tumours are the Wnt and MAPK pathways [3]. The MAPK pathway is activated by a receptor tyrosine kinase. Activating mutations of molecules, such as RAS, RAF or ERK, related to these signalling pathways, can produce tumour cell proliferation. In CRC, MAPK can be constitutively overactive. This type of hyperactivation can occur through three main signal transduction pathways: usually overexpressed MAPK/ERK pathway, also known as the RAS/RAF/MEK/ERK pathway; MAPK14 (p38 MAPK) pathway; activation of the stress-activated protein kinases/c-Jun Nh(2)-terminal kinase (SAPK/JNK) signalling pathway. The overactive state in the MAPK signalling cascade is determinant to influence the responsivity degree of the patient treated with EGFR inhibitors [4]. Moreover, p38MAPK signaling enhances glycolysis in cancer cells through the up-regulation of the glucose transporters [5] that can trigger cellular stress responses. Dysfunctions of the metabolic signaling pathways in the colonic epithelium and local immune cells are highly intertwined with the the gene/pheno-type dysfunction responsible of tumour progression and relatively antitumour immunity [6].

### 2-Warburg effect

In the colon, insulin resistance, hyperglycaemia and chronic inflammation reinforce the metabolic dysregulation associated with cancer development and promote the progression [7]. In fact, in this phase, the tumour cells proliferate rapidly by increasing their absorption of nutrients to satisfy their bioenergetic demands, developing a metabolism oriented towards anabolic pathways. Moreover, the nutrient-poor tumour microenvironment can influence the phenotype of macrophages that control both the innate and adaptive immunity response. In fact, a metabolic adaptation of tumour-associated macrophages (TAM) is triggered through the upregulation of the glycolytic genes linked to lactate derived from neoplasia which contributes to the polarization of tissue-associated macrophages (TAM) M2 which have an anti-inflammatory and pro-tumoural effect also in CRC [8, 9].

On the other hand, this reprogrammed metabolism, documented in many types of cancers is considered a hallmark of cancer cells and is called “Warburg effect”. The Warburg effect (WE) results in a glycolytic switch associated with oncogene activation, resulting in increased cytoplasmic production of ATP and lactate and disturbances in mitochondrial energy production [10, 11]. It is well known that CRC demonstrate the Warburg metabolic phenotype [12]. It is now evident that insulin resistance, hyperglycaemia and chronic inflammation lead to colon carcinogenesis through interaction with molecular pathways. The WE clearly suggests that the link between glucose metabolism, protein protonation state and the microenvironment of neoplasms, as resulting from interactions with the inflammatory infiltrate and related cytokine production, is a major actor of the development and progression of tumours [13, 14].

WE as the main characteristics of cancer can thus be triggered by infectious agents, followed by consequent effects such as genomic mutations [15], possibly switching benign adenomas to malignant carcinomas in the case of colorectal cancers. Inhibitors for mitochondrial ATP synthesis are being developed for colon cancer [16] in which the uptake of glucose is overregulated [17]. Moreover, tumor suppressor protein p53 was demonstrated negatively influences the oncogenic metabolic adaptation of cancer cells reverting WE [18].

### 3-Oxidative stress

Recently, it was also shown that some cancer cells use mitochondria, not glycolysis to generate ATP [19]. Such a cancer phenotype has been classified as the reverse Warburg effect (RWE). The underlying mechanism is based on a pathological collaborative interaction in tissue [20]. In particular, loss of stromal Caveoline-1 (Cav-1) expression results in increased production of nitric oxide, increased reactive oxygen species (ROS) production, increased oxidative stress, and mitochondrial dysfunction occurring in fibroblasts (supporting cells) which supply energy-rich metabolites to a cancer cell with fully functional mitochondria. Extensive morphological changes occur, these tumours are highly aggressive, fast growing, with a strong metastatic potential and poor outcomes for a patient. The reverse Warburg effect may exist in many different types of epithelial cancers [21] and the loss of stromal Cav-1 thus may be involved mechanistically in all the different phases of epithelial tumorigenesis.

CAV1 is frequently overexpressed in advanced colorectal tumors and it is implicated in enhanced aerobic glycolysis of tumor cells. Elevated CAV1 increases glucose uptake and ATP production by stimulating transcription of the glucose transporter GLUT3 via a high mobility group A1 (HMGA1)-binding site within the promoter [22]. HMGA1 chromatin remodeling protein is known as an important factor required by cancer cells for tumor progression and acquisition of a stem-like state [23, 24]. However, HMGA1 is also a ligand of RAGE (Receptor for advanced glycation end products) with direct implications in metabolic and molecular signaling dysfunction in the colon cancer microenvironment [25].

## AGES AND RAGES IN COLORECTAL CANCER

### 1-AGEs

The methylglyoxal (MG) produced through the glycolysis, is a highly reactive dicarbonyl compound and a major precursor of the cell advanced glycation end products (AGE) [26]. The AGE are highly reactive molecules, formed endogenously or exogenously during metabolic oxidative stress and accumulate in tissues normally with age [27]. Advanced glycation endproducts (AGEs) derive nonenzymatically from metabolism through glycation reaction between proteins, lipids, or nucleic acid with free reducing sugars [28].

AGEs can result from Maillard reaction or from glycolysis and oxidative pathways through the reaction between the electrophilic carbonyl group of reducing sugars with free amino group of proteins. Such reactions can generate unstable Schif’s bases that may form stable Amadori products after further rearrangements. Maillard in 1912 described the formation of melanoidins, a class of heterogeneous polymers that are formed when sugars and amino acids combine. In fact, Maillard products are synthesized trough a reaction between free amino groups of proteins, mainly arginine and lysine, and carbohydrates. This reaction was furtherly described by Amadori and is known as Amadori Heyns rear-rangement. In turn, the stable Amadori products undergo further modifcations, such as oxidation, dehydration or polymerization in the presence of transition metals to form more stable AGEs [29].

Many AGEs have been identifed such as the so-called GOLD (glyoxal lysine dimer), MOLD (methylglyoxallysine dimer), CEL (Nε-carboxyethyl-lysine), CML (pentosidine, and non-cross linking Nε- carboxymethyl-lysine).

The cellular functions of AGEs are mediated through binding to the receptor for advanced glycation end products (RAGE), a transmembrane molecule considered a pattern recognition receptor (PRR). It is a member of the immunoglobulin superfamily and a multi-ligand receptor that interacts with various ligands RAGE is thought to be associated with colon cancer metastasis and poor prognosis.

### 2-RAGE

RAGE is a multi-ligand signaling system highly expressed on the cell surface membrane of immune, endothelial or tumour cells when they are in the active phase. During embryonic development, there is a high RAGE expression especially in brain tissue. Its expression diminishes with the development and is almost absent in healthy adults. Only in healthy lung epithelium, does RAGE expression remain constitutive and relate to elevated levels of a unique isoform not found in other adult epithelia or cell types

RAGE consists in a 45–50 kDa molecule that binds to AGEs and other danger signaling molecules (DAMPs) to exert their pathophysiological roles in multiple disorders. These DAMPs include, among others, High-mobility group box proteins (HMGB1), S100 calgranulins and amphoterin.

Binding of RAGE with AGEs activates many signaling pathways also active in CRC, such as MAPK, NF-κB, PI3K, Akt, ERK1/2 and JAK-STAT, involved in inflammation, cell survival and cancer progression [30].

Hypoxia in cancer cells increases RAGEs expression and in cancer the involvement of the AGE-RAGE axis promotes the autophagic activity via the activation of autophagic proteins such as Beclin-1 with inhibition of apoptotic signaling. AGE-RAGE signaling also causes oxidative stress producing oxygen-free radicals, and activates NF-κB that causes secretion of pro-infammatory cytokines, growth factors, and adhesion molecules [31].

Studies conducted on knockout mouse models to understand the functions of RAGE, suggest that this receptor plays a regulatory role in sepsis [32]. In particular, it affects the homeostatic regulation of innate immunity, which in turn, can play a pivotal role in cancerogenesis [33].

**Figure 1:**
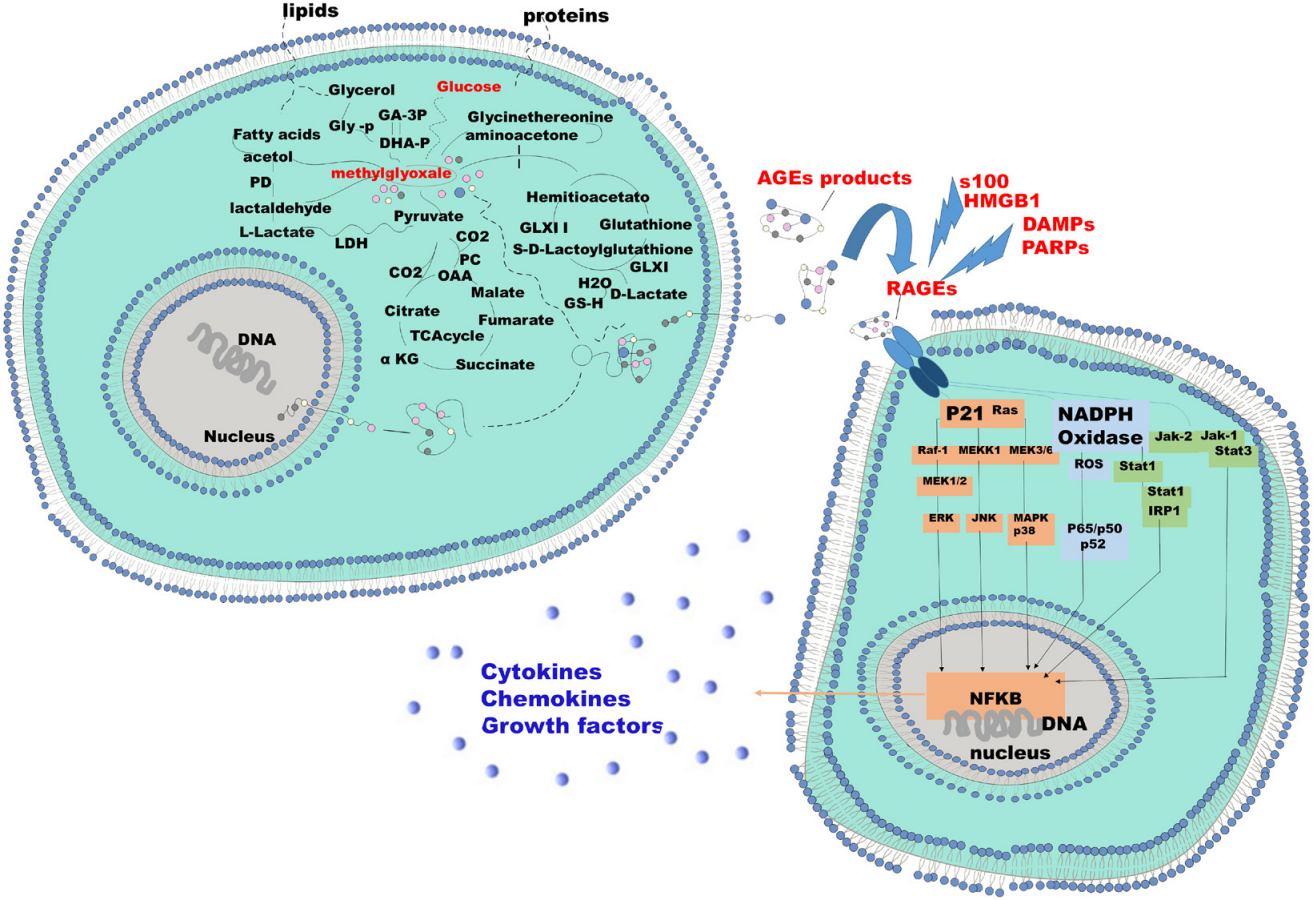
Mechanism of endogenous AGE formation and pathobiological actions of RAGE receptor ligands. The two cells represented here can be identified as a tumor cell and the stromal counterpart or vice versa. The energy demand of the tumor cell aimed at supporting the high proliferative standard that induces a proportional increase in the glyco-lytic pathway. The increased glycolysis results in an increased production of lactate and aldehydes such as glioxal and methylglioxal. These two aldehydes bind, by Maillards reaction, to the free radicals of the macromolecules, protein and non-protein present in the intracellular microenviroment resulting in adducts of Methylglyoxal, or advanced gly-cated adduct AGEs. These molecules gain extracellular space and bind to their receptors, RAGE. RAGE's expression is induced by the presence of the AGEs. RAGE activation results in the activation of downstream signaling pathways responsible for the release of cytokines, chemokines and growth factors. S-100 proteins DAMP and PAMP and HMBG in response to specific conditions share analogous ability to induce and bind RAGE.

When receptor ligand binding occurs, the resulting complex does not constitute a clear negative feedback signal aimed at inducing and regulating the clearance and degradation of the RAGE receptor. In fact, it has been shown that the receptor ligand complex instead functions almost as a positive feedback leading to a prolonged period of its expression and activation with consequent prolonged inflammation (Figure 1) [34]. This redundant positive effect created by the receptor ligand binding, results in an exasperated downstream molecular activation. This behaviour justifies why the pathways RAGE-dependent are involved in the pathogenesis of many simple and complex diseases, where inflammation is the least common. Currently, clinically relevant studies for RAGE associate its elevated plasma levels in inflammatory disease and brain injury with disease severity and functional outcome [35].

Among the countless diseases in which RAGE is involved there is also the onset and progression of colon cancer [36] (Table 1).

**Table 1:** Principal RAGE-ligands and activated pathways involved in the pathogenesis and development of colon cancer

Axis RAGE-ligand	Downstream Activated Pathways	Cancer phases	Pleiotropic effects	Study
**RAGE-AGEs**	*NFkB, ERK, MAPK*	Tumorigenesis and progression of colon cancer	*cell proliferation, inflammation, cancer progression*	Malara N, et al. [14] Liliensiek B [39]
**RAGE-S-100**	*NFkB, MAPK, IL-6, TNFα, TIL*	Advanced colon cancer	*cancer progression*	Onyeagucha BC, et al. [37] Sun X, et al. [53] Wang HY, et al. [55]
**RAGE-HMGB1**	*NFkB, TIL, PD-1*	Resistant colon cancer	*inflammation, cancer progression, immunosuppresion*	Yao X, et al. [71] Huang CY, [65]
**RAGE-DAMPs/PAMPs**	*SAPK/JNK, CdC42/Rac, p38MAPK, ERK1, CXCL2, IL-1, TNFα,*	Tumorigenesis and progression of colon cancer	*inflammation, cancer progression*	Escamilla-Tilch M, et al. [75] Stephens M, et al. [76]

### 3-AGEs-RAGE axis *in vitro*

Studies in human colon cancer cell lines have shown how the AGEs-RAGE axis induces the progression of cancer cells through the regulation of specific pathways, such as upregulation of carbohydrate response element binding protein (ChREBP) in the cell line HCT 116 (Human, colon, carcinoma). This protein, ChREBP, by promoting the enhancement of anaerobic glucose metabolism, drives the metabolic switch and suppression of p53. These actions combine to promote the growth and progression of cancer cells [37, 38]. Furthermore, in human colon cancer cell lines, Caco-2 and COLO320, the AGEs-RAGE axis increases ERK and MAPK and NF-ΚB respectively [39, 40]. The expression of Sp1 is also regulated by the binding of RAGE, with the AGEs through the activation of the ERK pathway. Sp1 is a key regulator of the molecular process that drives metastasis. This molecular actor intervenes in the modification of the stroma by promoting the degradation of type IV collagen with activation of metalloproteinases (MMPs). The prolonged NF-ΚB activation promotes tumour progression in colon cells through the inhibition of proapototic pathways that inhibit caspase activity [41–43].

## RAGE LIGANDS IN CRC

### S100 and RAGEs in colonrectal cancer

The S100 family of proteins is an important group that is part of the RAGE ligands. S100 proteins bind Ca2 + and their function is to regulate the levels of intracellular Ca2 + and consequently also of numerous Ca2 + signaling pathways. Unlike calmodulin and troponin-C, proteins linked to calcium metabolism and whose activities are limited to the intracellular environment, several proteins of the S100 family act as intracellular regulators but also as extracellular signaling proteins. They can be secreted and/or released to regulate the activities of target cells. Within the cell, S100 proteins exhibit a somewhat specific distribution. In the intracellular context, S100 proteins are involved in the regulation of proliferation, differentiation, apoptosis, Ca2 + homeostasis, energy metabolism, inflammation and migration/invasion. They interact with a variety of target proteins including enzymes, cytoskeletal subunits, receptors, transcription factors and nucleic acids. As extracellular signaling molecules, S100 proteins regulate cell proliferation, differentiation, survival and migration in normal and pathological conditions, inflammation and tissue repair and/or exert antimicrobial activity [43, 44]. Indeed, at the gene level, numerous S100 genes are specifically inducible by appropriate growth factors, cytokines and toll-like receptor (TLR) ligands. To fulfill the function of alarmins, or cell signaling molecules, they are secreted and may function as damage-associated molecular pattern factors that primarily mediate the functions of the innate and adaptive immune systems. Operationally, they stimulate the locomotion of tumour cells and/or participate in tissue repair. The S100B protein is expressed in astrocytes, some neuronal populations, Schwann cells, melanocytes, chondrocytes, adipocytes, skeletal myofibers and associated satellite cells, some dendritic cell and lymphocyte populations and a few other cell types. This protein acts as a stimulator of cell proliferation and migration and as an inhibitor of apoptosis and differentiation [45].

Cancer cells also exhibit a distinctive S100 protein profile which can be both stage-specific and subtype specific. In gliomas, S100B expression correlates positively with proneuronal, neuronal and classical subtypes, but not mesenchymal, whereas S100A8 and S100A9 expression correlates positively with mesenchymal subtypes [46, 47].

Extracellular protein S100A9, but not S100A8, binds to the EMMPRIN receptor and requires TNF receptor-associated factor 2 of adapter protein (TRAF2) to upregulate the expression of TNFα, IL-1, IL-6 and other factors [48]. Another example is given by the S10012 protein which mediates pro-inflammatory effects through binding to RAGE and the toll-like receptor such as TLR-4. The duple stimulation of RAGE and TLR-4s leads to the activation of the transcription nuclear factor NF-κB. Nuclear transfer of NF-κB induces inflammatory response and leukocyte recruitment. [47].

Furthermore, through RAGE receptors, under stress conditions, S100B, triggers NF-ΚB and MAPK signaling and stimulates the release of pro-inflammatory cytokines, such as IL6, TNFα and IL-1β [49, 50].

S100A8, S100A9 and S100P are other S100 proteins related to AGEs/RAGE progression mechanism of colorectalcarcinoma. They trigger many of the pathways above cited such as ERK, NF-kB [51, 52].

Interestingly, the membrane protein S100A16 and its main partner S100A14 and S100A4 are expressed by tumour cells in CRC and is associated with prognosis, indicating that the expression of these S100 proteins is a favorable prognostic biomarker and a therapeutic target.

The S100A4 protein also exhibits both intra- and extracellular activities. The expression of S100A4 in cells causes apoptosis, cell migration and maintenance of stem cells and in the extracellular space activates various processes by stimulating pro-inflammatory pathways and the expression of various molecules, such as cytokines [53]. In S100A4 knock out (S100A4 -/-) mice the expression of inflammatory cytokines and the recruitment of macrophages and neutrophils decreased significantly. On the other hand in wild type (WT) mice the effects, favouring colitis development, promoted by S100A4 could be abolished by a receptor for advanced glycation end products (RAGE)-specific inhibitor (FPS-ZM1) [54].

In summary, some effects of S100 proteins in CRC may be mediated by the regulation of TIL. Tumour-infiltrating lymphocytes (TILs) are associated with the host's immune status and are an important prognostic factor in many malignancies. Many reports have shown that an elevated level of TIL is a favorable biomarker in the prognosis of colorectal cancer. RAGE and its ligands are of fundamental importance in the modulation of TILs. RAGE and its ligands can play a fundamental role in the regulation of the lymphocyte infiltrate in CRC [55] by modulating its intensity and persistence in the tumour microenvironment (Table 1).

### HMGB1 and RAGEs in colonrectal cancer

HMGB1 is a chromatin binding factor in the nuclei of cancerous and normal cells, it plays a role in DNA repair, transcription, differentiation, extracellular signaling and somatic recombination [56]. The function of HMGB1 takes place by binding non-specifically to a smaller groove of the DNA and thus modulates the interaction of the DNA with transcription factors [57]. It enhances the activity of p53, p73, the retinoblastoma protein, transcription factors such as the Rel/NF-κB family and the estrogen receptor [58]. ERK/MAPK pathway is upregulated by HMGB1 in colon carcinoma [59]. In cell culture studies HMGB1 may show DNA damage caused by chemotherapy [60]. In colon cancer, HMGB1 and its receptor, are indicators of progression [61]. In an immunohistochemical study, HMGB1 overexpression was determined as 55.6% in relation to tumour invasion, lymph node status, distant metastasis and stage of colon cancer disease [62]. c-IAP2 is an antiapoptotic protein which may be upregulated through NFk-B activation via HMGB1. There is a strong correlation between upregulation of the apoptosis repressing HMGB1 and c-IAP2 proteins in the pathogenesis of colon carcinoma [63]. Upregulation of MMPs with HMGB1 has been associated with cancer cell proliferation of colon through the RAGE/Snail/NF-κB signalling pathways accompanied by the activation of MMP-7 [64].

HMGB1 expression was also correlated with overall survival and proved to be an independent predictor of worse prognosis in CRC cases [65] (Table 1). High expression of HMGB1 in the cytoplasm of breast cancer cells has been associated with high histological grade, pT stage and abundant TIL [66]. In patients with locally advanced rectal cancer and with elevated cyto-HMGB1 and PD-1 + TIL they showed better results. The best outcome is probably due to the release of HMGB1, which stimulates the maturation of dendritic cells (DCs) through the activation of TLR4, and the subsequent recruitment of TIL into the tumour [67]. Another explanation of the best outcome related the elevated cyto-HMG is due the inverted relationship with the CAV1 expression. In colon calcer cells CAV1 depleted cytoplasmic levels of HMGA1 are concomitantly increased. Increased expression of CAV1 stimulates HMGA1 interaction with the HMGA1-binding site in the SLC2A3 promoter at nuclear levels to improve the GLUT3 expression [68]. When GLUT3 is highly expressed in colorectal cancer (CRC) it is negatively linked to CRC patient outcome. GLUT3 expression protects CRC cells by energy stress in the tumour microenvironment to withstand nutrient scarcity and to exacerbate the malignancy of CRC cells [69].

### PAMPs/DAMPs and RAGEs in colonrectal cancer

The community of the microbiota in the lumen of the colon requires a careful system of surveillance of the intestinal immune system. In fact, the intestinal microbiota is a crucial biomodulator of the development and function of immune cells. Gut bacteria are directly involved in homeostatic regulation, function and differentiation of T cells. This is a saprophytic and mutually convenient contribution to both parties. In fact, while the microbes obtain a habitat and nourishment from the host, they return the favor by regulating the alimentary digestion and the protective immunity against the pathogens of the host [70]. Not surprisingly, in the early stages of the neoplastic transformation of the colonic epithelium there are phenomena of dysbiosis of the intestinal microbiota and increased intestinal permeability associated with inflammation. These same phenomena also underlie the tumour progression of CRC. Gut microbe-derived signals have been recognized to tune immune cells interacting with key players mediating host and microbe communication, known as pattern recognition receptors (PRRs). These signalling molecules, therefore considered to be part of the alarmine’s system, are expressed by innate immune cells such as dendritic cells (DC), monocytes/macrophages and natural killer (NK) cells [71]. RAGE, as an important pattern recognition receptor (PRR), may play a fundamental role in maintaining microbiological homeostasis within the colonic epithelium and how it can be considered as a perfect sensor for environmental stimuli. RAGE, as described above, is expressed on macrophages, smooth muscle cells, endothelial cells, epithelial cells. In addition to PAMP, RAGE also recognizes the molecular patterns associated with damage (DAMP) that induce inflammation [72]. The detection of PAMP and DAMP by RAGE drives a cascade of signals that converge to the nuclear factor κB (NF-κB) [73]. The interaction of ligands with RAGE activates a positive feedback loop by upregulating multiple signalling pathways such as SAPK/JNK (stress-activated protein kinase/c-jun-NH2-terminal kinase), Cdc42/Rac, p38 MAPK (protein kinase activated by mitogen) and ERK1/2 (Ras-extracellular kinase regulated by signal 1/2), which results, once again, in the increase of the transcription factor NF-KB and consequent production of cytokines, adhesive molecules and MMP (metalloproteinase of the matrix) [74]. Cytokines and chemokines that act as growth factors and promote angiogenesis have been associated with the development of polyps. In mice with defects in colon barrier integrity, bacterial invasion and increased expression of several inflammatory factors such as IL-17, Cxcl2, TNF-α and IL-1 can be observed [75, 76] (Table 1). In these proinflammatory responses, the mitochondrial dysfunction within the cancer cells, favors the excess reactive oxygen species release that can exacerbate the production of AGEs fueling the fire of inflammation [77].

## RAGE/AGES CROSS-TALK IN IMMUNE STATE AND IMMUNOTHERAPY OF COLON CANCER

K-Ras is a small nucleotide-binding molecule of guanine that cycles between the active (GTP-bound) and inactive (GDP-bound) forms [78]. Mutations of the oncogene in K-Ras cancel its interactions with GTPase activating proteins (GAP) with a consequent decrease in the hydrolysis rate of GTP. Furthermore, the oncogenic mutation confers constitutive activity to K-Ras leading to prolonged but not permanent activity which returns to baseline in the absence of continuous external stimulation [79]. It has been shown that during the initial stages of neoplastic transformation of the colon the oncogen Kras requires not only external stimuli but also for the continuous maintenance of the signaling necessary to support the growth of tumour cells [80]. The high levels of K-Ras activity generate inflammatory stimuli and oxidative stress, NFκB activation, COX2 expression and DNA damage leading to the release of HMGB1 and nucleic acids. These events form a Ras/inflammation feed-forward loop.

In this scenario RAGE is a central key player involved in maintaining the Kras/inflammation feed-forward loop. The role in chronic colon inflammation and tumour promotion of upregulation dependent on RAGE and its potential ligands S100a8 and S100a9 [81, 82]. Furthermore, bone marrow chimera experiments revealed that RAGE expression on immune cells maintains the inflammatory reaction during tumour promotion [83]. However, the importance of RAGE in tumours expressing the Kras oncogene may be even stronger. Animals expressing oncogenic Kras but deficient in RAGE expression showed a delay in cancer development. Mice deficient in the RAGE ligand, S100A9, show a significant reduction in the incidence and burden of colorectal cancers associated with colitis [84].

Colon cancer is known to generate a variety of RAGE ligands, including HMGB1 and S100 molecules, suggesting that while oncogenic K-Ras itself may be “non-pharmacological,” it may be possible to inhibit or modulate its role in cancer by blocking the critical feed-forward mechanism in which RAGE plays an important role. In addition to its previously described intra-nuclear role, HMGB1 functions as an extracellular signaling molecule by mediating different responses and binding to different receptors, including RAGE and toll-like receptors (TLRs) −2 and 4. Consequent are pleiotropic effects, such as proliferation cellular, differentiation, death, inflammation and immunity. When HMGB1 is passively released from dying tumour cells following chemotherapy in colorectal cancer, HMGB1 facilitates autophagy following cytotoxic insults for chemoresistance via its RAGE receptor via the MEK/ERK signaling pathway. ERK mediated phosphorylation of Drp1 is necessary to resist chemotherapy cytotoxicity as it promotes the induction of autophagy on surviving tumour cells to promote regrowth. Patients with highly phosphorylated Drp1 proteins are associated with an increased risk of developing relapse lesions after neoadjuvant chemoradiotherapy treatment in locally advanced rectal cancer [85].

Furthermore, there are cumulative points of evidence towards a cooperative interaction in the host immune response between RAGE and members of the Toll-like receptor (TLR) family. The question relating the possible physical association between RAGE and TLR remains to be address. RAGE and TLR shared three ligands, HMGB, S100 and bacterial cell wall component, LPS. The mechanism or the physicochemical conditions underlying the choice of ligands to bind preferentially RAGE or TLR is not well understood. Increasing evidence in literature support their potential synergism on down-stream signaling pathways and the outcome of these interactions on the inflammatory response [86].

TLRs are found on the cell surface or intracellular in endosomes. They have well-established roles in the host immune response to infections and their activation normally contributes to the induction of protective immune responses [87].

TLR can be recognized by exogenous and endogenous ligands (DAMPs), as low-molecular-weight hyaluronic acid, fibrinogen, fibronectin, heparan sulfate peptidoglycans, and AGEs. AGE/TLR interaction determines the expression of NFκB with an increase in adhesion molecules (ICAM and VCAM, which lead to the recruitment of macrophages and neutrophils) and production of TNF, IL-1α, IL-4, IL -10, Cytokines IL-12 MCP-1, PAI-1 and tPA. [88]. TLRs represent a molecular link between the phenotype of inflamed tumour cells, the regulation of T cell activation and antitumour immunity and can modulate tumour progression and chemoresistance. The experimentally induced absence of the three receptor forms of TLR (TLR 3,7,9) results in tumour regression dependent on both CD4 and CD8 T cells and protects mice from subsequent tumour provocation. [89].

The CRC can evade immune surveillance in various ways, for example through the recruitment of immunosuppressive cells such as regulatory T cells (Treg), the secretion of factors such as TGF-β or through the activation of Immune checkpoints. On the other hand, these tumors can activate, through the secretion of pro-inflammatory cytokines, cellular factors promoting carcinogenesis such as STAT3. Among the immunotherapies used against CRC are recombinant cytokines, agents modulating the immune microenvironment, oncolytic viral therapy, small molecules, autologous T cells, vaccines, antibodies against specific antigens and immune checkpoint inhibitors. Combination of immunotherapies for CRC are also tested.

Imumune checkpoint inhibitors are used in order to treat CRC because they provide a prolonged disease control and exhibit an acceptable toxicity degree. Patients with MSI have higher responses to PD-1 treatment [90].

In the interaction between cancer cell and the immune system, a central role has recently been attributed to the immunosuppressive action of PD-L1. Cancer cells can locally produce PD-L1 to inhibit immune cell surveillance. In this regard, it has been shown that PD-L1 is expressed on the surface of exosomes secreted by tumour cells, which are found in the bloodstream with the function of inhibiting the anti-tumour activity of T cells. It has been shown that there are numerous transcriptional regulators. which can induce the expression of PD-L1. In the tumour microenvironment, PD-L1 can be modulated by inflammatory cytokines such as IFNγ, which induces activation of STAT1 and increased transcription of PD-L1 [91, 92].

HIF1, Myc, STAT3, AP-1, and NF-ÎºB have also been shown to increase PD-L1 transcription in response to a variety of upstream signals. PD-L1 expression is significantly elevated in colon cancer tissues. During chemotherapy HMGB1 is passively released and through its RAGE receptor, upregulating the MEK/ERK signaling pathway increases NF-ΚB transcription and indirectly stabilizes PD-L1 [93]. Indeed, NF-ΚB, by upregulating the CSN5 deubiquitinase, inhibits the ubiquitination and proteasomal degradation of PD-L1 in tumour cells [94, 95]. Intriguing is the consideration that the RAGE-ligand axis can promote an immunosuppressive microenvironment by activating the PD-1/PD-L1 checkpoint, together with other previously reported immunosuppressive cytokines, such as IL4, IL10, PGE2 and TGFβ [96]. In this scenario, it is possible to imagine that possibly significantly blocking PD-1/PD-L1 could enhance CD8 + T-cell-mediated antitumour immunity against cancer cells. Therefore, a treatment with anti-PD-1 blocking antibodies to increase the number of CD8 + memory T cells in the tumour microenvironment may be useful as an immune prevention approach to antagonize the immune evasion of tumour cells [97–99].

## CONCLUSIONS

**Figure 2:**
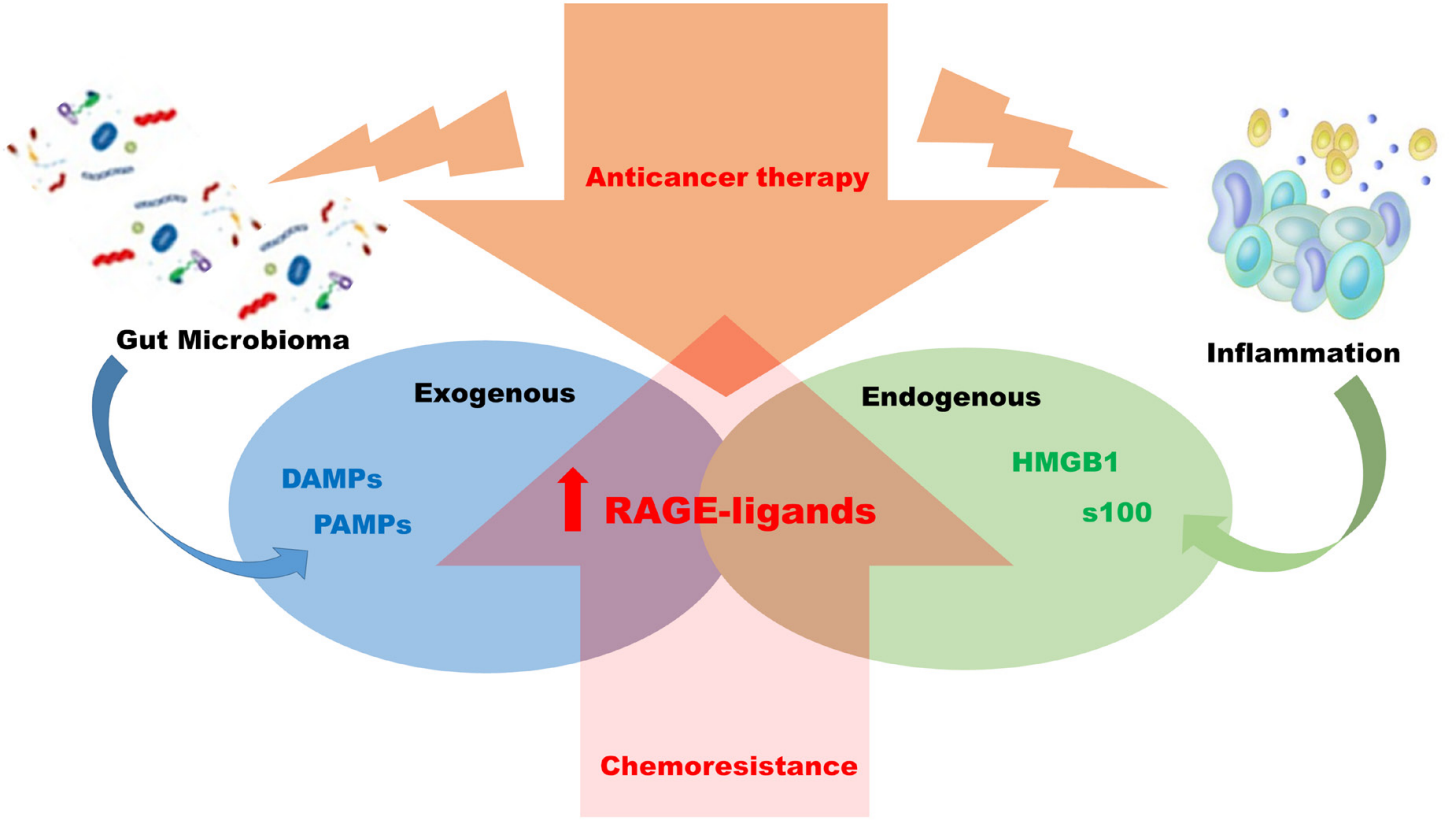
Mechanism of chemo resistance RAGE-ligands dependent. Anticancer treatments in colon rectal carcinoma, chemotherapy and radiotherapy have as a side effect the induction of local inflammation and alteration of the intestinal flora. The two biological phenomena can be associated with the induction of RAGE-ligand axis with consequent activation downstream of pro- inflammatory pathways favoring chemo resistance establishment.

**Figure 3:**
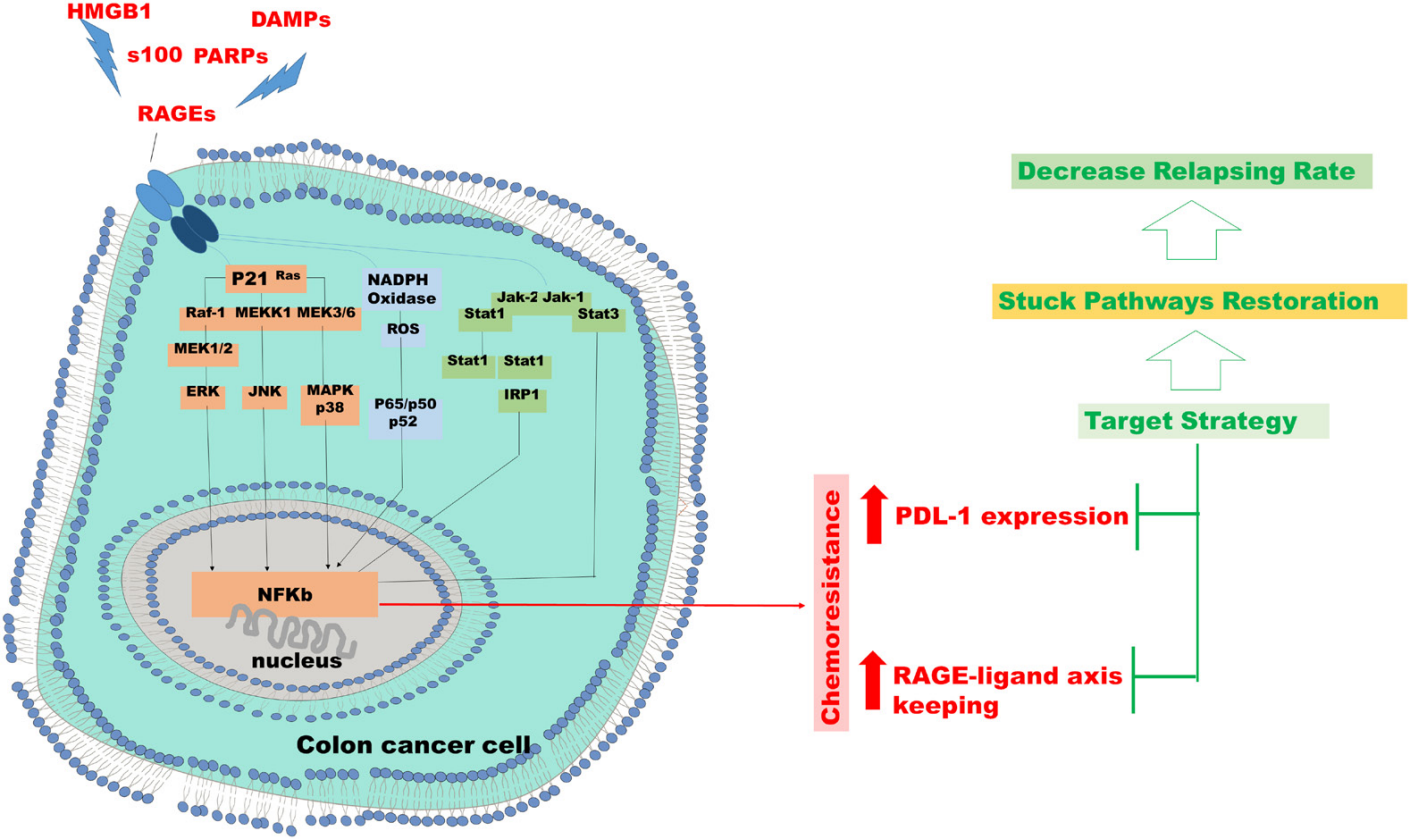
Chemo resistance in colon cancer cell. Hypothesis of targeted therapeutic intervention in resistant cases in which the RAGE or PDL-1, or both, overexpression is proven in colon cancer cells.

The RAGE-ligand axis intervenes and supports the tumour progression of the CRC by inducing and activating molecular pathways promoting chemoresistance and immuno-escape phenomena. In particular, endogenous ligands as molecules produced and present in the intracellular microenvironment, which, once released in the extracellular space, become signalling molecules aimed at the endogenous activation of the RAGE. These molecules, AGEs, S100, HMGB1, are all connected to pro-cancerogenic molecular pathways of the colon and are also produced during chemotherapy. Their presence and concentration in the extracellular space induces and influences the activity of the RAGE receptor on cellular surface membrane of tumour cells, stromal cells and of immune cells. Moreover, exogenous microbial agents, relative to disturbance phenomena on colon microbiome, releasing DAMPs and PAMPs free to interact with RAGE, contributed to its constitutional state of activity. Metabolism and metabolites conditioned the microenvironment by modulating the immune response and on the other hand, the concomitant activation of RAGE, by inducing the indirect increasing of the transcriptional activity of NFKB, affects the production and release of cytokines and chemokines. Cytokines and chemokines are essential actors in the inflammatory scenario that implies the progressive evolution of the chemo resistant CRCs (Figure 2). The initial presence of the K-Ras mutation, which guides the choice of first-line treatment for CRCs, loses consistency on the molecular cross-linked front of the advanced tumour. We hypothesize that in the case of advanced CRCs the therapeutic action of cancer containment should be concentrated by working on several molecular fronts. Without disregarding the target therapy but supporting it to favour the persistence and duration of a positive response, through direct inhibition of RAGE-ligand axis and PDL-1expression, previously verifying their sharing of the activity. From the data emerging in this review, patients with chemoresistant CRCs cannot be retrained only through the presence or absence of K-ras mutation, evaluated by i.e., cfDNA analysis [100, 101], but it is necessary, through the analysis of markers such as RAGE or PDL-1 expression, through immunocytochemical approach [102–104] on i.e., circulating tumour cells [105–107], to verify how to support the possible choice of a targeted therapy or in the absence of this, to identify an optional and personalized therapeutic intervention (Figure 3).

## Abbreviations

CRC: Colorectal cancer
AGEs: Advanced glycation end products
RAGE: Receptor for advanced glycation end products
PRR: Pattern recognition receptor
CIN: Chromosomal instability
MSI: Microsatellite instability
APC: Adenomatous Polyposis Coli
MMR: Mismatch repair genes
CIMP: CpG island methylator phenotype
MAPK: mitogen-activating protein kinase
CMS: Consensus Molecular Subtypes
VEGF: Vascular endothelial growth factor
EGFR: Epidermal growth factor receptor
SAPK/JNK: Stress-activated protein kinases/c-Jun Nh(2)-terminal kinase
TAMs: Tumour Associated macrophages
TILs: Tumour-infiltrating lymphocytes
WE: Warburg effect
ADP: Adenosine diphosphate
G-6-P: Glucose 6-phosphate
LDH: Lactate dehydrogenase
PDK: Pyruvate dehydrogenase kinase
DAG: Diacylglycerol
MG: Methylglyoxal
KG: α-ketoglutarate
GLXI: Glyoxalase I
GLXII: Glyoxalase II
GS-H: Glutathione
DHAP: Dihydroxyacetone phosphate
GA-3P: Glycerol-3-phosphate dehydrogenase
ROS: Reactive oxygen species
NF-kB: Nuclear factor kappa B
JAK: Janus kinase
JNK: c-Jun N-terminal kinase
MAPK: Mitogen-activated protein kinase
MEK: Mitogen-activated protein kinase
ERK: Extracellular signal-regulated kinase
STAT: Signal transducer and activator of transcription
DAMPs: Damage-associated molecular pattern molecules
S100B: S100 calcium-binding protein B
HMGB1: High mobility group box 1 protein
PAMPs: Pathogen-associated molecular patterns
ChREBP: Carbohydrate response element binding protein
DCs: Dendritic cells
NK: Natural killer
HCT 116: Human, colon, carcinoma
MMPs: Matrix metallopeptidases
TLR: Toll-like receptors
PDL-1: Programmed death-ligand 1
